# *Anacardium* Plants: Chemical, Nutritional Composition and Biotechnological Applications

**DOI:** 10.3390/biom9090465

**Published:** 2019-09-09

**Authors:** Bahare Salehi, Mine Gültekin-Özgüven, Celale Kırkın, Beraat Özçelik, Maria Flaviana Bezerra Morais-Braga, Joara Nalyda Pereira Carneiro, Camila Fonseca Bezerra, Teresinha Gonçalves da Silva, Henrique Douglas Melo Coutinho, Benabdallah Amina, Lorene Armstrong, Zeliha Selamoglu, Mustafa Sevindik, Zubaida Yousaf, Javad Sharifi-Rad, Ali Mahmoud Muddathir, Hari Prasad Devkota, Miquel Martorell, Arun Kumar Jugran, Natália Martins, William C. Cho

**Affiliations:** 1 Student Research Committee, School of Medicine, Bam University of Medical Sciences, Bam 44340847, Iran; 2 Department of Food Engineering, Faculty of Chemical and Metallurgical Engineering, Istanbul Technical University, Maslak, Istanbul 34469, Turkey; 3 Department of Gastronomy and Culinary Arts, School of Applied Sciences, Özyeğin University, Çekmeköy, Istanbul 34794, Turkey; 4 Bioactive Research & Innovation Food Manufac. Indust. Trade Ltd., Katar Street, Teknokent ARI-3, B110, Sarıyer, Istanbul 34467, Turkey; 5 Laboratory of Applied Mycology of Cariri, Department of Biological Sciences, Cariri Regional University, Crato 63105-010, Ceará, Brazil; 6 Laboratory of Planning and Synthesis of Drugs, Department of Antibiotics, Federal University of Pernambuco, Recife 50670-901, Pernambuco, Brazil; 7 Laboratory of Microbiology and Molecular Biology, Department of Biological Chemistry, Regional University of Cariri, Crato 63105-010, Brazil; 8 Department of Agronomy, SAPVESA Laboratory, Nature and Life Sciences Faculty, University Chadli BENDJEDID, El-Tarf 36000, Algeria; 9 State University of Ponta Grossa, Departament of Pharmaceutical Sciences, Ponta Grossa 84010-330, Paraná, Brazil; 10 Department of Medical Biology, Faculty of Medicine, Nigde Ömer Halisdemir University, Campus, Nigde 51240, Turkey; 11 Department of Food Processing, Bahçe Vocational School, Osmaniye Korkut Ata University, Osmaniye 80500, Turkey; 12 Department of Botany, Lahore College for Women University, Jail Road, Lahore 54000, Pakistan; 13 Zabol Medicinal Plants Research Center, Zabol University of Medical Sciences, Zabol 61615-585, Iran; 14 Department of Horticulture, Faculty of Agriculture, University of Khartoum, Shambat 13314, Khartoum North, Sudan; 15 School of Pharmacy, Kumamoto University, 5-1 Oe-honmachi, Chuo-ku, Kumamoto 862-0973, Japan; 16 Program for Leading Graduate Schools, Health Life Science: Interdisciplinary and Glocal Oriented (HIGO) Program, Kumamoto University, Kumamoto 862-0973, Japan; 17 Department of Nutrition and Dietetics, Faculty of Pharmacy, University of Concepción, Concepcion 4070386, Chile; 18 Universidad de Concepción, Unidad de Desarrollo Tecnológico, UDT, Concepcion 4070386, Chile; 19 Govind Ballabh Pant National Institute of Himalayan Environment and Sustainable Development, Garhwal Regional Centre, Srinagar-246 174, Uttarakhand, India; 20 Faculty of Medicine, University of Porto, Alameda Prof. Hernâni Monteiro, 4200-319 Porto, Portugal; 21 Institute for Research and Innovation in Health (i3S), University of Porto, 4200-135 Porto, Portugal; 22 Department of Clinical Oncology, Queen Elizabeth Hospital, Hong Kong SAR, China

**Keywords:** *Anacardium*, cashew nut, antioxidant, antimicrobial, industrial applications, food preservative

## Abstract

*Anacardium* plants are native to the American tropical regions, and *Anacardium occidentale* L. (cashew tree) is the most recognized species of the genus. These species contain rich secondary metabolites in their leaf and shoot powder, fruits and other parts that have shown diverse applications. This review describes the habitat and cultivation of *Anacardium* species, phytochemical and nutritional composition, and their industrial food applications. Besides, we also discuss the secondary metabolites present in *Anacardium* plants which display great antioxidant and antimicrobial effects. These make the use of *Anacardium* species in the food industry an interesting approach to the development of green foods.

## 1. Introduction

The Anacardiaceae family has about 77 genera and 700 species, mainly distributed in tropical, subtropical, and temperate areas [1,2]. Among them, the genus *Anacardium* has 20 species, widely distributed in tropical areas [3,4]. *Anacardium occidentale,* also known as cashew nut, is the most widely cultivated and used species. At which, *Anacardium microcarpum*, commonly known as Cajui, and *A. occidentale* are widely used for medicinal and nutraceutical purposes [5]. Therefore, a summary of the current research outputs on this genus is crucial in order to promote its proper use and identify the current scientific gaps to drive future research.

## 2. Habitat and Cultivation of *Anacardium* Species

*Anacardium* grows in stony, sandy, loamy and heavy soils at elevation around 600 m. It prefers well drained soil and cannot grow in nutritionally poor soils. These species show poor growth in heavy, waterlogged clay or saline soils [6]. *Anacardium* genus grows in pH ranging from 4.5 to 6.5. Trees are fast growing with a life span of 30–40 years; in their third or fourth year they begin to bear fruit. The root system of a mature tree consists of a tap-root and a well-developed, extensive network of lateral and sinker roots, after grown from seed [7]. Production usually takes three years after planting, and eight years before economic yield can begin. However, some breeds, like the dwarf cashew tree, starts production in only one year and attains economic harvest in three years [8]. The pollination of flowers is done by flies, bees, ants, and wind. The plant is self-fertile, prefers moist soil, can tolerate drought, strong wind, but not maritime exposure [9].

Plants are not frost tolerant, and prefer a pronounced dry season of 3–4 months [10]. Plants produce their best crops when grown in their favorable climatic conditions. In semi-arid tropical areas of Africa, India, Sri Lanka, and southeastern Asia cashew nuts are cultivated commercially. In 2010, total world production of cashew nuts was 3.6 million tons, harvested from 4.4 million hectares. The leading producer of commercially sold cashew apples is Brazil [11].

The wild cashew (*Anacardium excelsum*), generally considered indigenous to the northern part of South America, is actually cultivated in the coastal region of India, mainly in states like Maharashtra, Goa, Karnataka, Kerala, Tamil Nadu, Andhra Pradesh, Orissa, Madhya Pradesh, West Bengal, northeastern states, Andaman and Nicobar Islands [12]. *A. occidentale* is a tough drought-resistant tropical and subtropical tree. It is an evergreen tree growing 10–15 m high with a short, irregular shaped trunk [13]. It is being planted on 1200 hectares of land in Pahang. A survey concluded that 120,000 hectares of land in Peninsular Malaysia are appropriate for its planting. This tree is irregularly a shrub with resin canals. The very young cashew apple is green or purple, and later turns green. When ripe, the apple becomes red or yellow, or a mixture of both. The cashew tree has a rigorous lateral root system and a tap-root which penetrates deeply into the soil [14]. *A. othonianum* is a tree native from the tropical savanna region of Brazil. Its fruit is similar (but smaller than) to the common cashew tree (*A. occidentale*) of the Brazilian Northeast. In the wild, the adult tree ranges from 2 to 6 m (3 m on average), and produces from 200 to 600 fruits every season. 

## 3. Nutritional Composition

Studying the proximate, mineral and functional attributes of defatted and undefatted cashew kernel flours, it was found that in defatted cashew, the kernel flour proximate content of protein, crude fibre and carbohydrate (34.0, 6.2 and 32.2%, respectively) is significantly higher than that of undefatted cashew kernel flour [15]. The proximate, mineral, and energy profiles were also studied in dried cashew nut testa [16]. The crude protein (190 g/kg), fibre (103 g/kg), fat (20.1 g/kg) and ash contents (20.2 g/kg) of dry matter were detected along with metabolizable energy of 7.12 MJ/kg dry matter. The moisture content, ether extracts (crude fat) and total ash (4.4, 1.6 and 1.8%, respectively) were found to be decreased in defatted flour. Likewise, noticeable variations were recorded in all the studied mineral elements between the defatted and undefatted flours, besides manganese, which showed significantly higher contents in undefatted samples compared to defatted ones. However, no significant variations were detected in bulk density, foam capacity/stability, emulsion capacity and nitrogen solubility (pH 8) between defatted and undefatted flours samples. Other parameters, like water/fat absorption capacity, emulsion stability and nitrogen solubility of these two samples at pH 8.0 also displayed significant variations [15].

Eleven samples of raw cashew kernel (*A. occidentale*) collected from India, Brazil, Ivory Coast, Kenya, Mozambique, and Vietnam were investigated for the total dietary fibre, sugar, protein, lipid profile, sodium, and energy contents [17]. Total fat comprises the major component corresponding to 48.3% of the total weight, of which 79.7% were unsaturated fatty acids, 20.1% saturated fatty acids, and 0.2% *trans* fatty acids. Proteins (21.3 g/100 g) were the second major constituents followed by carbohydrates (20.5 g/100 g). The mean value of sodium content was 144 mg/kg. The mean energy content was 2525 kJ/100 g.

The alterations in physicochemical properties of the juice of yellow and red cashew apples varieties from Yamoussoukro (Ivory Coast) were evaluated by Adou, et al. [18]. The protein content ranged from 0.51 to 0.53 g/100 g and major amino acids in order of size were leucine, cysteine and asparagine. Glucose, fructose and sucrose concentrations (g/L) between the varieties ranged from 47.2 to 65.8, 100.7 to 110.3 and 2.5 to 5.3, respectively. Among the organic acids, citric acid was found in the majority (290.7 and 1092.1 μg/mL), followed by tartaric acid (497.5 to 693.3 μg/mL), acetic acid (48.2 to 266.5 μg/mL), oxalic acid (197.8 to 204.3 μg/mL) and fumaric acid. The pH of the juice ranged from 4.37 to 4.5 while titratable acidity was 0.5 to 0.85%. Similarly, the total soluble solids (10.2 to 10.9%), dry matter (7.8 to 10%) and ash (1.3 to 1.9%) contents also varied among the samples. The vitamin C content varied between 370.9 and 480.3 mg/100 g while total sugars were found between 162.7 to 168.1 g/L in two studied varieties [18].

### 3.1. Amino Acids

Ion-exchange chromatography was used to evaluate the amino acids composition of *A. occidentale* (Table 1), in order to enhance the quality of cereal protein through food complementation [19]. *A. occidentale* possessed a total amino acids of 659.17 mg/g protein, and glutamic acid was present in the highest concentrations. Total essential amino acid percent was 51.0% in the species, and total acidic amino acids were 30.4%. The calculated isoelectric points for *A. occidentale* were 3.9, displaying they can all be precipitated at acidic pH. Threonine was detected as the limiting amino acid in *A. occidentale.* Likewise, the percentage of cystine concentration in total sulphur amino acid was 50.5%. In the Rico, Bullo and Salas-Salvado [17] study, based on *A. occidentale,* the amino acid with highest presence was glutamic acid with 4.60 g/100 g, whereas the one with lower presence was tryptophan with 0.32 g/100 g. In accordance with the study performed by Fagbemi [20], the major dominant amino acid was glutamic acid (183.5–214.0 mg/g crude protein) while tryptophan (3.9–9.2 mg/g crude protein) and leucine (34.8–38.2 mg/g crude protein) were the limiting amino acids.

### 3.2. Vitamins and Minerals

Looking at the nutritional composition, few vitamins (B, C, and E; Table 2) and minerals (Na, K, Ca, Mg, P, Fe, Cu and Se; Table 3) have been identified in *Anacardium* plants [23]. The concentrations of four hydrophilic vitamins in the fruit of red fruited species of *A. occidentale* were found as: ascorbic acid 34.2 mg/100 g, thiamine 15.5 mg/100 g, riboflavin 2.90 mg/100 g, and niacin 0.23 mg/100 g [24]. In the Rico, Bullo and Salas-Salvado [17] study, based on *A. occidentale*, vitamin E was the most abundant vitamin with an average contribution of 5.80 mg/100 g. In a study by Tamuno and Onyedikachi [15], cashew apple juice pasteurized at 80 °C for 15 min was packaged in diverse packaging materials like green, brown, white bottles and polyethylene sachet in 200 mL batches and kept at room (30 °C) and refrigeration (4 °C) temperatures for four months to study the effect of packaging materials on both the vitamin C content and pH of cashew-apple juice. Juice stored at 30 °C exhibited significant differences in vitamin C content (48–159 mg/100 mL) and pH (5.0–6.2) from the juice stored at 4 °C. Maximum loss of vitamin C was recorded for samples in polyethylene sachet (83–48) from the first to fourth month, respectively. However, no significant impact of bottle color on vitamin C loss was recorded as the values ranged between 169–128 mg/100 g (white), 187–130 mg/100 g (green) and 188–132 mg/100 g (brown) from the first to the fourth month of refrigeration.

### 3.3. Lipids and Fatty Acid Profile

High-yielding varieties of cashew were evaluated for lipids in cashew kernel (Table 4). It was found that neutral lipid from kernel contributed 96% of the total lipids while the remaining 4% was contributed by glycolipid and phospholipid. Unsaturated fatty acids like oleic and linoleic acid were found in a higher majority in triglycerides, while saturated fatty acids like lauric and myristic were the dominant glycolipids. Varietal difference was noticed with respect to the composition of neutral and glycolipids. However, no variations were detected in the composition of phospholipid among high-yielding varieties [29]. *A. occidentale* nut samples were processed by drying, boiling, fermentation, germination and roasting. The oils extracted from nuts were studied for fatty acid composition. The study revealed that the proximate composition of the nuts was significantly influenced by the processing techniques. Oleic acid (57.9–66.8%) and linoleic acid (10.4–17.7%) were found to be the major unsaturated fatty acids. Palmitic acid (8.9–11.7%) and stearic acid (6.9–8.4%) were identified as the saturated fatty acids [20]. In the Rico, Bullo and Salas-Salvado [17] study, based on *A. occidentale,* 14 fatty acids were detected, and oleic acid was dominant, contributing to 60.7% of the total fat, followed by linoleic (17.77%), palmitic (10.2%), and stearic (8.93%) acids.

### 3.4. Polysaccharides

*A. occidentale* gums from Brazilian plants were found to have higher galactose and lower arabinose and rhamnose concentrations when compared to cashew gums from India and Papua [33]. However, the distribution of other compounds, like glucose, mannose and glucuronic acid was similar (Table 5). Gel permeation chromatography detected the presence of 6% polysaccharide-protein complex, 42% polysaccharide of *M_pk_* 1.6 × 10^4^ in cashew gum.

### 3.5. Antinutrients and Heavy Metals

Methanol (80%) extract of the inner stem bark of *A. occidentale* was quantitatively evaluated for antinutrients and few heavy metals [34]. Several compounds like tannins (5.75%), oxalates (2.50%), saponins (2%), phytate (0.25%) and cyanide (0.03%) were also recorded. Iron from dried crude (8.92 mg/100 g) was recorded from the extract whereas lead and cadmium were absent in the extract.

## 4. Phytochemical Composition of *Anacardium* Plants: A Brief Overview

**Figure 1:**
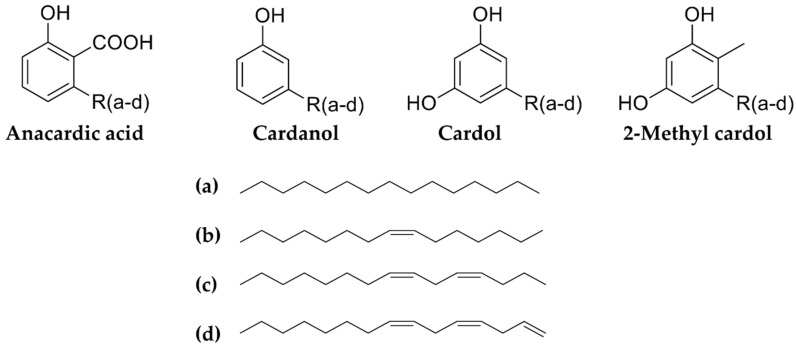
Chemical structures of anacardic acids, cardanols, cardols and 2-methyl cardols.

Studies have been performed to investigate the chemical composition of *Anacardium* species, and over 170 phytochemicals have been recorded (Table 6, Table 7, Table 8 and Table 9). Cashew nut shells comprise the main agro-waste produced from cashew nut processing factories and contain about 30–35 wt % oil or cashew nut shell liquid. A mixture of four potential compounds, anacardic acid, cardanol, cardol and 2-methyl cardol, have been found in this liquid (Figure 1). The components of cashew nut shell liquid are converted into industrially important chemicals through several reactions and have shown to be promising renewable resources for synthesizing various industrial chemicals [35]. In addition, cashew nut shell liquid is a cheap and renewable agro byproduct, consisting of some versatile monomers. Solvent-extracted cashew nut shell liquid contains over 80% anacardic acid with more than 90% degree of unsaturation in its C15 side chain. For instance, from anacardic acid monomer have been synthesized anacardanyl acrylate and anacardanyl methacrylate monomers [36].

*A. occidentale* leaf and shoot powder comprise the most commonly assessed *Anacardium* plants for its chemical composition using gas chromatography (GC), atomic absorption spectrometry (AAS) and high-performance liquid chromatography (HPLC). Both the leaf and shoot powder of the species exhibited adequate content of dry matter, crude protein, β-sitosterol and stigmasterol. Powdered samples also have saturated fatty acid and monounsaturated octadecenoic, and gadoleic in larger amounts. A significant amount of trace minerals has also been detected. In addition, significant variations have been found depending on the extraction methods. For example, ether extract of the species did not exhibit the presence of alkaloids, while coumarins and quinones were recorded in both ethanol and aqueous extracts. Likewise, ethanol extracts also have prominent amounts of anthocyanidins, triterpenes or steroids and tannins. Flavonoids and saponins have been detected in aqueous and ethanolic extracts [31].

On the other hand, the currently available methods also affect in differential ways the final chemical composition. Conventional shelling methods, like oil-bath roasting, drying, direct steam roasting, and open pan roasting along with a novel “Flores” hand-cracking method have been used to determine the levels of bioactive compounds present in cashew nut kernels. Among them, *β*-carotene (9.57 μg/100 g dry matter), lutein (30.29 μg/100 g of dry matter), zeaxanthin (0.56 μg/100 g of dry matter), *α*-tocopherol (0.29 mg/100 g of dry matter), *γ*-tocopherol (1.10 mg/100 g of dry matter), thiamin (1.08 mg/100 g of dry matter), stearic acid (4.96 g/100 g of dry matter), oleic acid (21.87 g/100 g of dry matter), and linoleic acid (5.55 g/100 g of dry matter) have been recorded in considerable amounts in raw cashew nut kernels. However, a significant reduction in carotenoids, thiamin, and unsaturated fatty acids levels has been stated in cashew nuts using all conventional shelling methods mentioned, whereas similar contents of these compounds were observed using Flores hand-cracking method [4]. Sixteen high-yielding varieties of *A. occidentale* were already characterized for their chemical composition using cashew apples and kernels samples. No significant variations were detected in starch, amino acids, protein, and sodium contents in cashew kernels among the high-yielding varieties. Further, the content of reducing sugar was negligible in the kernel compared with total sugar. However, the levels of vitamin C, amino acids, phenols and tannins in high-yielding varieties cashew apple demonstrated significant variations. A small amount of non-reducing sugars has also been recorded in cashew apples. For example, while M 6/1, Bla 256-1, M 10/4 and M 44/3 varieties possess low tannins, seeming to be better for apple juice extraction, the qualitative composition of sugars, organic acids and phenols did not exhibit any variations among the varieties [26].

### 4.1. Phenolic Compounds

Nut shell liquid derived from commercial *A. occidentale* comprises anacardic acid, cardol, and cardanol as the major phenolic constituents. Each of these phenolic constituents is heterogeneous, and possesses saturated, monoene, diene, and trienes in the fifteen-carbon side chain (Table 6).

Several isolation methods have been described for the isolation of anacardic acid, cardol, and cardanol for industrial applications. Calcium anacardate is used to selectively isolate anacardic acid. Briefly, the acid-free cashew nut shell liquid is treated with liquor ammonia followed by a hexane/ethyl acetate (98:2) extraction to isolate the mono phenolic component, cardanol. Then, on ammonia solution is extracted with ethyl acetate/hexane (80:20) to obtain cardol [39]. Likewise, phenolic compounds from cashew nuts and testa have been investigated for determining their roasting impact. Cashew testa has a higher extract yield, total phenolic content, and proanthocyanidin contents in soluble as well as in the bound fractions, when compared with the whole nuts and kernels. Among the phenolic acids detected in both samples are syringic, gallic, and *p*-coumaric acids. Similarly, flavonoid compounds, like (+)-catechin, (-)-epicatechin, and epigallocatechin, have also been recorded. However, their concentrations increase with increasing temperature. Thus, these findings suggest that roasting at high temperatures and for short periods of time effectively increases the chemical constituents of cashew nuts and testa [56]. However, the phenolic composition of cashew nuts and testa was also investigated after roasting, by subjecting samples to treatments of low and high-temperatures. Results showed that roasting enhances the total phenolic content in both soluble and bound extracts from whole nut, kernel and testa, and decreased proanthocyanidins besides the soluble extract of cashew kernels [56].

The cashew apple juice was found to possess gallic, protocatechuic and cinnamic acids (free and conjugate). Cinnamic acid was obtained in the hydrolyzed form on cutting followed by storage at 40 °C. However, the 5-hydroxymethylfurfural concentration in cashew apple juice was found to increase after injury and storage at higher temperatures, indicating non-enzymatic browning [44]. *A. occidentale* ethyl acetate phase was also investigated for its phenolic composition using HPLC, being demonstrated the presence of catechin, epicatechin, epigallocatechin and gallic acid [42]. *A. occidentale* immature and ripe peduncles obtained from orange (CCP 76) and red-colored (BRS 189) clones were also used for preparation of juice or fibrous fraction followed by ultra-performance liquid chromatography–tandem mass spectrometry (UPLC-MS) analyses. Simultaneously, the soluble fraction of the species was subjected to enzymatic evaluation. It was shown that ripe juice samples from both cashew clones possess cinnamoyl glucoside, while immature juice samples contain monogalloyl diglucoside and digalloyl. Although the activity of UDP-glycosyltransferases was found to vary between clones, and cinnamoyl glucoside was derived as its product, it was suggested to be a possible chemical marker of ripe juice samples from both clones. The highest specific activity of enzyme flavonol synthase was recorded in both cashew clones and its product, and a positive activity was detected in both immature and ripe stages [57]. Methanol, hexane and ethyl acetate extracts of *A. occidentale* shoots were investigated for the total phenolic content using Folin–Ciocalteau assay. The methanol extract had seven-fold higher total phenolic content than the hexane and ethyl acetate extracts [58]. The hydroethanolic extract of *A. occidentale* leaves was also studied for its chemical composition. Phenolic compounds were identified as the major components of the extract. Liquid chromatography combined with mass spectrometry revealed the presence of glycosylated quercetin, amentoflavone derivative and a proanthocyanidin tetramer, with a total amount of phenolics in the extract of 35.5% and flavonoid content of 2.58% [45].

Anacardic acids, cardanols and cardols content were also assessed in cashew apple, nut (raw and roasted) and cashew nut shell liquid. Major alkyl phenols and anacardic acids were found at higher amounts (353.6 g/kg) in cashew nut shell liquid, and were lowest (0.65 g/kg) in roasted cashew nut samples. To highlight that, although cashew apple and fibre comprise exclusively anacardic acids, cashew nut shell liquid also possesses an abundance of cardanols and cardols. Moreover, raw and roasted cashew nuts also contain hydroxy alkyl phenols at low concentrations. Alkyl phenol classes are separated from the basic fractionation of cashew nut shell liquid. Individual anacardic acids, dominant cardanols and cardols were also purified from cashew nut shell liquid fractions using semi-preparative HPLC and detected through nano-ESI-MS-MS, GC/MS and NMR analyses. The isolation of cardanol from toxic cardol from cashew nut shell liquid was performed. A mixture of methanol and ammonium hydroxide (8:5) was used to dissolve technical cashew nut shell liquid followed by hexane extraction to obtain cardanol, and the obtained methanolic ammonia layer was isolated with a mixture of ethyl acetate and hexane to yield cardol [41]. Similarly, *A. excelsum* extracts and fractions were investigated using GC with a mass selective detector, being revealed the presence of few phenolic compounds, such as 2-(1,1-dimethylethyl)-4-(1,1,3,3-tetramethylbutyl)phenol [49].

Phenolic lipids, namely 2-(8″Z-eicosenoyl)-6-(8′Z-pentadecenyl) salicylic acid, 2-(9″Z-hexadecenoyl)-6-(8′Z,11′Z-pentadecadienyl) methyl salicylate, 2-(10″Z, 13″Z-nonadecadienoyl)-6-(8′Z, 11′Z-pentadecadienyl) salicylic acid, 2-(16″Z-pentacosenoyl)-6-(8′Z-pentadecenyl) salicylic acid and 2-(9″Z-octadecenoyl)-6-(8′Z, 11′Z-pentadecadienyl) methyl salicylate along with three known constituents, cardols, anacardic acid and cardanols, which have also been extracted from cashew nuts, and MS and NMR spectroscopic methods have been used to determine the structures of these compounds [59].

#### 4.1.1. Flavonoids

Various flavonoids have been isolated from *Anacardium* species. For example, occidentoside, a new biflavonoid-C-glycoside, also named (-)-salipurposide, and β-sitosterol have already been separated from *A. occidentale* nut shells. Some spectral and chemical evidences have been used to establish the structure of occidentoside as tetrahydroninokiflavone-C-glucoside (III) form. This is the first biflavonoid, consisting of one flavanone and one chalcone unit, and also the first C-glycoside in the biflavonoid series. The presence of (-)-salipurposide was also reported in cashew nut shells [60].

In general, both the identification and quantification of flavonoids in cashew apple have been performed using liquid chromatography, with diode array detection and electrospray ionization mass spectrometry (LC-DAD-ESI/MS) methods. Methanol: water extract of the species had one anthocyanin and 13 glycosylated flavonols. A comparative assessment with standards or positively detected flavonoids in cranberry revealed the presence of 3-*O*-galactoside, 3-*O*-glucoside, 3-*O*-rhamnoside, 3-*O*-xylopyranoside, 3-*O*-arabinopyranoside and 3-*O*-arabinofuranoside of quercetin and myricetin, as well as kaempferol 3-*O*-glucoside. The anthocyanin identified was 3-*O*-hexoside of methyl-cyanidin. However, in the hydrolyzed extract, trace amounts of delphinidin and rhamnetin were also recorded, suggesting the presence of their glycosides, but unidentified in the original extract [47]. The ethanolic extracts of cashew leaves have also been fractionated and separated using HPLC. The biflavonoid agasthisflavone was also extracted from one of these four fractions obtained from cashew nut [61]. *A. occidentale* leaves and stem samples have also been screened for other phytochemicals. 

#### 4.1.2. Tannins

The extraction of tannins from *A. occidentale* has been performed under the effect of particle size, temperature, methanol content and time using a pressure autoclaving method. Both qualitative and quantitative estimations of tannins have been performed using spectrophotometric assays, and the impact of each extraction conditions determined in terms of total tannins. Temperature has been considered the main factor affecting tannin extraction. Preliminary analyses revealed that small particle sizes have the potential to achieve high extraction efficiency, though fine grinding was not found suitable due to pressure drop, grinding energy and product purification costs. Indeed, tannin extraction is enhanced by a particle size range of 5–15 mm. Less noticeable reduction/degradation of these compounds was detected at low temperatures (40 °C). However, the effect of temperature on the extraction of these compounds is markedly higher than the effect of time and substrate concentration [62].

Tannins were extracted from the skin and flesh of four cashew apple genotypes collected from Brazil and Bénin (West Africa) using acetone/water (60:40, *v/v*) along with separation from monomeric phenols. Acid-catalyzed degradation of tannins was performed using phloroglucinol and their products were studied by HPLC–DAD/ESI–MS. High percentages of (-)-epigallocatechin and (-)-epigallocatechin-*O*-gallate, followed by minor quantities of (-)-epicatechin and (-)-epicatechin-3-*O*-gallate were detected in both skin and flesh tannins. All the compounds were found in the 2,3-*cis* configuration. Half of the skin sample tannins were galloylated (∼20%) as compared to flesh tannins [54]. Other studies have also been performed to analyze the average molecular weight, main functional groups and thermal properties of tannins isolated from *A. occidentale,* using HPLC, FTIR and thermogravimetric analysis. A comparative analysis of results was made using tannic acid. The bands characteristic of CvC, CZC and OH bonds were exhibited by FT-IR spectra [63]. Likewise, the average molecular weight, main functional groups and thermal properties of tannins isolated from *A. occidentale* were investigated using HPLC, FT-IR and thermogravimetric analysis. Bands characteristic of C=C, C-C and OH bonds were identified through FT-IR spectra analysis [63]. Tannin content was also investigated in the testa obtained from high-yielding varieties of cashew and from samples of commercial cashew processors in India and compared with almond testa. Varieties and commercial samples exhibited significant variations in cashew testa composition. Almond testa possesses a lower tannin content than the cashews, being therefore proposed to be useful for developing food or feed additives [64].

### 4.2. Anacardic Acids

Natural cashew nut shell liquid comprises anacardic acids as major constituents. It is obtained by the extraction of cashew shells with hexane at room temperature. The major constituents of cashew nut shell liquid are anacardic acid and salicylic acid derivatives (monoene–15:1, diene–15:2, and triene–15:3) [65].

Purification and characterization of 1-*O*-*trans-*cinnamoyl-beta-d-glucopyranose isolated from cashew apple juice were also performed. Although it was absent in the immature green stage, its concentration increased at the turning and is even higher at the mature ripe stage (up to 6.2 mg/100 g of fresh weight, f.w.). Cinnamoyl glucoside ester in cashew apple genotype was accumulated in the epidermis and was 4–5 times higher than in the flesh, reaching 85 mg/100 g of f.w. for skin of the Brazilian clone EMBRAPA 50. Whole cashew apple comprises 6 to 20 mg of 1-cinnamoyl glucose/100 g [54]. Another study was performed to isolate anacardic acid from natural cashew nut shell liquid using supercritical carbon dioxide. The solubility data for natural cashew nut shell liquid in supercritical carbon dioxide under varying conditions of pressure (100, 200, and 300 bar), temperature (40 and 50 °C), and CO_2_ flow rate (5, 10, and 15 g/min) were also obtained. The best conditions with supercritical carbon dioxide were 50 °C and 300 bar at a flow rate of 5 g/min CO_2_. Under these supercritical CO_2_ conditions and from a cashew nut shell liquid sample (3 gm), high purity anacardic acid was isolated from crude natural cashew nut shell liquid (82% of total anacardic acid) within 150 min. Different spectroscopic methods (UV-vis, FT-IR, and (1)H NMR) and HPLC analysis of anacardic acid extracted by supercritical carbon dioxide indicated that the anacardic acid isolated by supercritical carbon dioxide had a higher quality compared to that obtained through a conventional method involving several chemical conversion steps [66]. Resorcinolic lipids from *A. occidentale* nut oil extract was also detected from bacterial and graminaceous sources and liposomal structures. Those vesicular structures exhibited higher entrapment of the marker and size stability [67].

### 4.3. Carotenoids, Carotenoid Esters, and Anthocyanins

Yellow, orange, and red peeled *A. occidentale* apples were studied for their pigment profile. A total of 15 carotenoids and carotenoid esters were detected (Table 6). Among them, β-carotene, and β-cryptoxanthin palmitate were the most dominant compounds in peels and pulp of all samples. Orange-peeled pulp samples (2.2 mg/100 g f.w.) were found to possess higher total carotenoid contents than the yellow and red-peeled cashew apples (0.69–0.73 mg/100 g f.w.). Color variations between the yellow and red colored samples revealed the presence of a non-carotenoid pigment type in red peels. Among the four identified anthocyanins, 7-*O*-methylcyanidin 3-*O*-β-d-galactopyranoside was the main anthocyanin detected using NMR spectroscopy. The presence and absence of anthocyanins were mainly determined by the red and yellow peel color, whereas the enhanced amount of carotenoids mainly by the orange appearance of the peel. Therefore, a rich source of provitamin A (ca. 124 μg retinol-activity-equivalents/100 g pulp, f.w.) was exhibited by orange-peeled fruits [53].

### 4.4. Essential Oils, Volatiles and Aroma Compounds 

A large number of studies have reported the essential oil and volatile constituents in *Anacardium* species (Table 7 and Table 8).

Leaves, fruits, and flowers of the red and yellow *A. occidentale* varieties were studied to isolate essential oils using hydrodistillation followed by GC/MS analysis. (*E*)-*β*-ocimene (28.8%), *α*-copaene (13.6%), and *δ*-cadinene (9.1%) were dominant in the leaves oil from the red variety. On the other hand, palmitic (19.6%) and oleic (19.6%) acids were major compounds in the fruit oil of the red variety, whereas in the case of oil from the yellow variety, palmitic acid (11.4%), furfural (10.0%), 4-hydroxydodecanoic acid lactone (8.2%), (*E*)-hex-2-enal (7.2%), (*Z*)-hex-3-enol (6.2%), and hexadecanol (6.2%) were the dominant ones. *β*-caryophyllene (26.0%), methyl salicylate (12.8%), and benzyl tiglate (11.3%) were detected as the major constituents in the flowers oil of the red variety [68]. Fresh leaf oil of *A. occidentale* investigated using GC and GC/MS exhibited the presence of 51 components representing 99.3%. Limonene was detected as the major compound (85.9%) followed by other major components, like β-caryophyllene (1.7%), α-pinene (1.5%), α-terpineol (1.1%) and α-ylangene (1.0%). In the leaf oil, forty-six other compounds were also detected, which were not reported earlier in the leaf oil [78]. Similarly, the essential oil isolated from *A. occidentale* fresh leaves using steam distillation method demonstrated a yield of less than 0.1%. A total of 182 constituents were detected using GC and GC/MS analysis. β-phellandrene + limonene (17.5%), methyl chavicol (11.4%), germacrene B (8%), *trans*-α-bergamotene (7.9%), germacrene D (5.9%), β-copaene (5.9%), linalool (5.9%), α-cadinol (5.9%), β-caryophyllene (5%), 9-epi-(*E*)-caryophyllene (5%), β-phellandrene (4.9%), α-phellandrene (4.8%), hexadecanoic acid (4.7%), epi-α-cadinol (4.5%), β-bisabolene (4.4%), and epi-α-muurolol (4.1%) were identified as the main components in the essential oil of the species [69]. In addition, the heating impact on the *trans* isomerization of edible oils obtained from cashew nut shell liquid was also evaluated. Edible oils were treated with varying heating temperatures and different time intervals. Results showed a suppression of *trans* fatty acids formation and stimulation of conjugated linoleic acid (CLA) isomers synthesis on adding the appropriate concentration of cashew nut shell liquid to edible oils during heating. Formation of *trans*-oleic, *trans*-linoleic, and *trans*-linolenic acids isomers was inhibited by 0.2% concentration of technical cashew nut shell liquid, while formation of 9 t,11 t-CLA and 10 t, 12 t-CLA isomers was increased at same dose. No significant decrease was recorded in the acid value in the presence of 0.2% technical cashew nut shell liquid in corn oil, but a significant reduction was observed in the peroxide value. The trans isomerization of natural cashew shell liquid exhibited better function compared to vitamin E and tertiary butylhydroquinone, suggesting that it can be an effective additive in edible oils [30].

The thermal concentration of cashew apple juice resulted in a considerable loss of volatile compounds, which ultimately damaged product quality. The aroma volatiles evaporated on thermal concentration of cashew apple juice were recovered using a water phase. Volatiles from the water phase were isolated using dichloromethane, concentrated under nitrogen flow, using GC/MS. The Osme GC-olfactometry technique was used to analyze each volatile in the cashew aroma analyzed by five trained judges. A total of 71 volatiles were detected, among them 47 were found to be odor active. Alcohols, particularly heptanol, *trans*-3-hexen-1-ol and 3-methyl-1-butanol, were recovered preferentially from the cashew water phase and imparted green grass and fruity aroma from the water phase. Esters, namely, ethyl 2-hydroxyhexanoate, ethyl *trans*-2-butenoate and ethyl 2-methyl butanoate, represented 21% of the total chromatogram area and were found to be responsible for the fruity/cashew-like aroma of the water phase. In contrast, volatile 3-methylbutanoic and 2-methylbutanoic acids exhibited the greatest odor effect in the GC effluents of the water phase [79].

Pasteurized and reconstituted (from concentrate) Brazilian cashew apple nectars (a secondary product from the production of cashew nuts) were investigated for their aroma volatiles by GC/MS and GC-O/GC-FID. Several compounds, like methional, (*Z*)-1,5-octadien-3-one, (*Z*)-2-nonenal, (*E*,*Z*)-2,4-decadienal, (*E*,*E*)-2,4-decadienal, β-damascenone, and δ-decalactone were identified in cashew apple products for the first time. The strongest aroma volatiles were butyric acid, ethyl 3-methylbutyrate, 2-methylbutyric acid, acetic acid, benzaldehyde, homofuraneol, (*E*)-2-nonenal, gamma-dodecalactone, and an unknown compound. A total of 36 aroma volatiles were identified in the reconstituted sample, while 41 compounds were detected in the pasteurized sample. However, both samples possessed 34 common aroma components. Moreover, ethyl 3-methyl butyrate and 2-methylbutyric acid showed characteristic effects in cashew apple (warm, fruity, tropical, and sweaty) [80]. Also, Indian, Vietnamese, and Brazilian cashews were evaluated for volatile constituents in both raw and roasted samples using selected ion flow tube-mass spectrometry. Based on color determination, the optimum times for roasting were also assessed. Color and volatiles of raw cashews roasted in oil for 3 to 9 min at 143 °C were determined. A significant correlation was stated between L* value and roasting time. Similarly, darkness increased with the increase in roasting time. Vietnamese, Indian, and Brazilian cashews exhibited the 6, 8, and 9 min as the optimum roasting time, respectively. Raw cashews possess a lower amount of volatiles as compared to roasted cashews. Most of volatile concentrations noticeably increased on roasting of Brazilian, Indian, and Vietnamese cashews. However, few volatiles significantly decreased during roasting. Brazilian cashews exhibited a significant decrease in ethanol and 1-heptene during roasting, while toluene was decreased in Vietnamese cashews. Brazilian cashews possess significantly higher concentrations for most volatiles as compared to Indian and Vietnamese cashews. No significant variations were recorded in the highest volatile concentration in Indian and Vietnamese cashews. A higher concentration of Strecker aldehydes, including methylbutanal, 2-methylpropanal, and acetaldehyde, was recorded in roasted cashews. There was also degradation of sugars in the form furan-type compounds, while lipids were oxidized to form alkanals, such as hexanal. The rate of color formation and the development of volatiles varied for the cashews studied from the three geographical locations [81].

The chemical composition of the volatile oils from five Anacardiaceae species was investigated using GC and GC/MS. (*E*)-Caryophyllene (31.0%) and α-pinene (22.0%) was the major component in *A. humile* leaves oil, while *A. occidentale* oil comprises (*E*)-caryophyllene (15.4%) and germacrene D (11.5%). Similarly, in case of *A. fraxinifolium* leaves oil, (*E*)-*β*-ocimene (44.1%) and α-terpinolene (15.2%) are the dominant constituents, whereas the *Myracrodruon urundeuva* oil has an abundance of δ-3-carene (78.8%). However, the oil from *Schinus terebinthifolius* leaves, collected during March and July, exhibited a variable chemical composition [71].

The comparative assessment of the technique performance on volatile extraction for the aroma/flavor of fresh cashew apple juice was performed using dynamic headspace analysis. Among the 181 compounds detected, 44 esters, 20 terpenes, 19 alcohols, 17 hydrocarbons, 15 ketones, 14 aldehydes were detected. Esters (*n* = 24) and terpenes (*n* = 10) were identified as the major aroma compounds, demonstrated by sensory evaluation of GC effluents. The four techniques were capable of isolating esters; however, the dynamic headspace methodology formed an isolate with greater concentration, which enabled both detection (GC/MS) and sensory evaluation of the chromatographic effluents. Dichloromethane was found to be most effective solvent for terpene extraction without further concentration. These two techniques also allowed the isolation of higher amounts of other volatiles important to the cashew aroma, like aldehydes and alcohols, also exhibiting a high potential [82]. A study was also conducted to assess the influence of lactic acid fermentation on volatile compounds from cashew apple juices using HS-SPME/GC/MS, combined with chemometric, with 81% of the variance explained by the first principal component. A decrease in ethyl acetate, ethyl-2-methyl butanoate, ethyl crotonate, ethyl isovalerate, benzaldehyde, and ethyl hexanoate in cashew juice was imparted by *Lactobacillus casei*-mediated fermentation. The measurements of both stability and formation of these compounds in cashew juice can thus be used as a volatile marker to follow juice fermentation [83]. In addition, some volatile organic compounds were investigated in typical Brazilian fruit and fruit juices by a solid phase microextraction method using a capillary GC system with flame ionization detection and GC/MS. More than 100 volatile organic compounds were detected in both fruit and fruit juices studied by GC/MS. This method was also employed to quantify ethanol, naphthalene and benzoic acid in four different species of Brazilian fruit and their juices [74]. Also, volatile extracts from *A. occidentale* were evaluated for their chemical composition. The study exhibited the presence of the terpenoids (*E*)-4,8-dimethylnona-1,3,7-triene (DMNT) and (*E*,*E*)-4,8,12-trimethyltrideca-1,3,7,11-tetraene (TMTT) [84].

### 4.5. Other Compounds

Four compounds, zoapatanolide A, agathisflavone, 1,2-bis(2, 6-dimethoxy-4-methoxycarbonylphenyl) ethane (anacardicin) and methyl gallate were isolated from *A. occidentale* samples using bioassay-guided fractionation, spectroscopy, Alamar blue fluorescence-based viability assay in cultured HeLa cells and microscopy [52]. In addition, both leaf and bark samples from 15 Anacardiaceae species collected from Mexico were assessed for their toxic phenol concentration, namely, catechols, resorcinols and bioflavonoids using paper and thin layer chromatography (Table 9). Catechols were found in the majority of species, while only a few species contained toxic resorcinols. With regards to bioflavonoids, the basal group in Anacardiaceae, namely, Spondias and allied genera do not contain bioflavonoids, although they were present in the rest of the Anacardiaceae genera [85].

In addition, using chromatographic methods, hexane extracts from *A. occidentale* bark were studied to isolate two hypoglycemic principles, namely, stigmast-4-en-3-ol and stigmast-4-en-3-one. These purified compounds were subjected to spectroscopic analysis to characterize their respective structures [46].

In a study, a comparative assessment of both red and yellow fruited species of *A. occidentale* leaves revealed high concentrations of alkaloids, whereas lower levels were found in the fruits of both species. Fruit and roots of the red fruited species, as well as the fruit and stem bark of the yellow fruited species displayed the lowest flavonoid contents. Fruit and leaves of the red and yellow fruited species showed higher concentrations of anthocyanins than the other plant parts. The content of saponins in the stem bark of the red fruited species was 10-fold higher than the concentration of saponins and 9-fold higher than that in yellow fruited species. However, hydrogen cyanide was not detected in both fruits and roots of both species. The leaves of both species exhibited the highest contents of phenolics. Stem bark of both species demonstrated the higher tannins concentration, while fruits displayed lower values [24]. The volatile fractions of two Brazilian honeys, known as caju and marmeleiro, were characterized by a column extraction technique. Totals of 59 and 36 volatile compounds were detected from caju and marmeleiro honeys, respectively. The major compounds detected in caju honey were furfuryl mercaptan, benzyl alcohol, *δ*-octalactone, *γ*-decalactone, eugenol, benzoic and isovaleric acids, phenylethyl alcohol, and 2-methoxyphenol; while in marmeleiro honey, only a few compounds, like isovaleric acid, *γ*-decalactone, benzoic acid, and vanillin, were identified as potent odorants.

## 5. Food Preservative Applications of *Anacardium* Plants

### 5.1. Food Preservation from Natural Sources

Foods are constantly subject to deterioration and oxidation, due to microorganism contamination (yeasts, bacteria and molds) and other causes, like insects, enzymatic reactions, thus causing loss of quality [95,96,97]. In addition, they are responsible for toxin production, which is harmful for consumers, generating serious foodborne diseases, that according to the World Health Organization (WHO), a global estimate showed around 420,000 deaths [95,98,99]. There are several types of foods, for example, fruits, meat, fish, beverages, dairy products that can be infected by a wide diversity of bacteria, such as *Escherichia coli, Listeria monocytogenes, Clostridium botulinum, Bacillus cereus, Salmonella* spp., *Campylobacter* spp., *Staphylococcus aureus, Shigella* spp., and so on [96,99,100,101].

On the other hand, and although these are important issues, people are looking for a more natural way of life with wellness and this can be provided through eating. In this sense, preservation is a very important step to maintain food quality, integrity and safety, to avoid microorganism spoilage and growth, thus increasing the foodstuff shelf life and stability. The concern regarding the use of synthetic food preservatives and antioxidants due to their undesirable side effects (toxicity, teratogenicity or carcinogenicity) for organisms is making further research emerge towards the search for components with a natural origin. These natural sources are able to improve the quality of human health and the environment [96,101,102,103].

There is a wide diversity of natural product classes that have been used as antimicrobial and antioxidant agents in foods. For instance, phenolic compounds (flavonoids, tannins, floroglucinols, polyphenols), organic acids, essential oils, consisting of monoterpenoids and phenylpropanoids, alkaloids, saponins, peptides and thiols are the most commonly used [97,99,100,104,105,106]. An increasing list of essential oils has been considered as Generally Recognized as Safe (GRAS) by the Food and Drug Administration (FDA) to be used as synthetic flavoring substances and adjuvants (e.g. thymol, linalool, eugenol, limonene, citral, vanillin, menthol, carvone, carvacrol, anetol) [96,99,105]. Therefore, plant-isolated metabolites may be conceived as excellent antimicrobial and antioxidant candidates, playing a key role as preservative agents in foods, in place of synthetic chemical agents, thus raising their standard quality, shelf life and, amplifying their use in the food industry, for fruits, meat, sausages and mayonnaise, which are industrially packaged and sold in grocery stores [97,103].

### 5.2. Anacardium Antioxidant and Antimicrobial Activities and its Potential as Food Preservative

**Figure 2:**
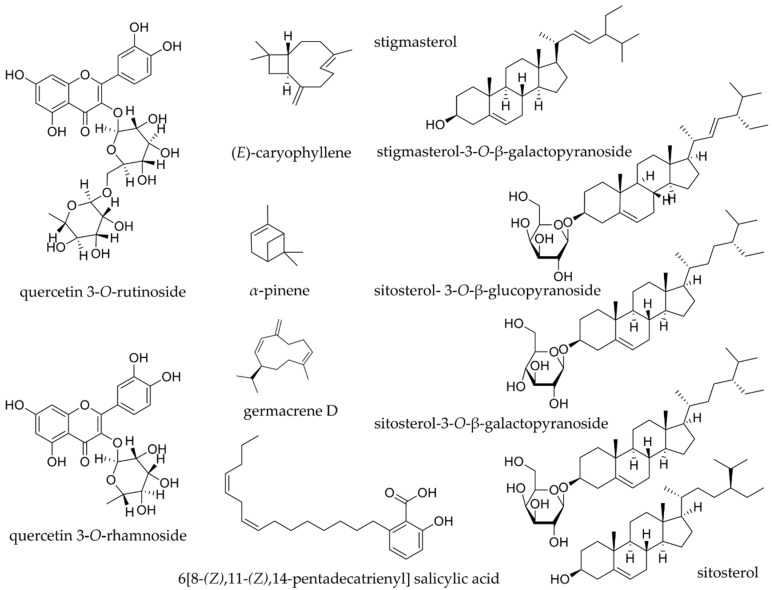
Chemical structures of *Anacardium* compounds with biological activities.

Several studies have shown the antimicrobial and antioxidant activities of *Anacardium* spp., the predominance of studies turn to the species *A. occidentale*, whether in its pure form or extracts and fractions; these features are able to preserve the food and to avoid the spoilage. In sequence will be demonstrated the data searched in databases such as Pubmed, Google Scholar, Scielo, Bireme and Portal de Periódicos Capes. In Figure 2 are represented the molecular structures of *Anacardium* compounds that have biological activities and are discussed in this section.

The cashew nut shell has a mixture of anacardic acids, representing 70% of its total chemical content, of which 6[8-(*Z*),11-(*Z*),14-pentadecatrienyl] salicylic acid is the main bioactive compound of the species *A. occidentale;* due the presence of this type of acid, the species has great antimicrobial potential [107,108]. The observation of non-viable growth after 28 days of Gram-positive and negative bacteria such as *S. aureus*, *Bacillus subtilis* and *E. coli* in tomato paste and tomato ketchup inoculated with 2 x 10^4^ cfu/g was caused by the anacardic acid tested at a concentration of 0.014%, suggesting it could be used as a natural food preservative [108].

Besides this, several studies have reported the antimicrobial potential of *Anacardium* species, for example, the study performed by Himejima and Kubo [109] reported the antimicrobial activity of sixteen phenolic compounds from the cashew nut shell oil and, most of them were active against *Breuibacterium ammoniagenes*, *E. coli*, *S. aureus* and *Streptococcus mutans* and *Propionibacterium acnes.*

The water and ethanol extracts of cashew nut shell waste inhibited *S. aureus* (3.13 μg/mL), *B. cereus* (3.13 μg/mL), and *Enterococcus faecium* (6.25 μg/mL), with minimal inhibitory concentration (MIC) cited. In addition to the antimicrobial test, the cashew nut shell waste extract (150 μg/mL) was tested to evaluated its antioxidant activity, which inhibited DPPH (75.5%) and ABTS (97.1%) and, when compared with the trolox, the values were calculated to be 57.1 and 56.2 μmol equivalent antioxidant capacity, respectively. Therefore, the authors concluded the cashew nut shell could have two uses, one is the use as a food preservative and the second is to reduce agricultural waste production [110].

The essential oil of *A. occidentale* clones was clinical tested against human pathogens and, the clone F-848 presented a notable activity against (*Acinetobacter anitratus* and *S. aureus*) with MIC ranging from 6250–12500 μg/mL and 6250–50000 μg/mL, respectively, and 3125 μg/mL for *Candida albicans* [111]; in this way, approaching another use but showing its ability to act against bacteria and fungi.

Antioxidant activity was also observed by isolated 6-pentadec(en)ylsalicylic acids from cashew *A. occidentale* nut and apple; they prevent the generation of superoxide radicals inhibiting enzymes, such as xanthine oxidase and soybean lipoxygenase-1, this last one inhibited by anacardic acid. As reported, there is a prooxidant inhibition of enzymes that are related to the production of reactive oxygen species and chelation of divalent metal ions (Fe^2+^ and Cu^2+^), without extinguishing reactive oxygen species [90].

The study performed by Trevisan, Pfundstein, Haubner, Würtele, Spiegelhalder, Bartsch and Owen [87] analyzed the antioxidant potential of different forms of cashew presentation, being the cashew apple, nut (raw and roasted) and cashew nut shell liquid, and assessed their ability to inhibit the enzyme xanthine oxidase and superoxide generation. The most efficient was cashew nut shell liquid with 100% of inhibition, and it should be mentioned that this fraction had higher quantities of anacardic acids besides containing cardanols and cardols. The hexane extract of cashew fibre also revealed a good ability, inhibiting 94%, while apple showed 53% of inhibition, both containing only anacardic acids. Interestingly, the mixture of anacardic acids presented an IC_50_ = 0.6 mM, with anacardic acid-1 (IC_50_ = 0.27 mM) being the most potent. Thus, they have shown higher antioxidant capacity than the cardols and cardanols (IC_50_ > 4 mM).

Ethanol extracts of leaves, stem bark and flowers of *A. occidentale* presented phenolic compounds, xanthones, triterpenes, flavones and anacardic and gallic acids. The extracts presented antioxidant activities in DPPH assay and, for the antimicrobial test, the ethanol extract of flower was the most active in comparison with the other extracts. For this assay, the extracts were evaluated in 14 microorganisms: *C. albicans*, *Candida tropicalis, Klebsiella pneumoniae*, *Helicobacter pylori*, *Salmonella choleraesuis*, *Pseudomonas aeruginosa*, *Proteus mirabilis*, *E. coli, S. mutans*, *Lactobacillus acidophilus*, *S. aureus*, Methicillin Resistant *S. aureus* (MRSA), *Enterococcus faecalis*, *Streptococcus pyogenes*, being effective against Gram-positive and negative bacteria and, fungi [112].

Flavonoids present in the ethyl acetate extract from leaves of *A. occidentale* (agathisflavone and a mixture of quercetin 3-*O*-rutinoside and quercetin 3-*O*-rhamnoside) showed that the mixture was the most effective in DPPH, TAC (total antioxidant capacity) and FRAP (ferric reducing antioxidant powder) assays. In the antimicrobial context, the mixture of compounds stood out again, being active against *P. aeruginosa*, *Clostridum sporogens*, *S. aureus*, *E. coli* and *K. pneumoniae* [51].

The cashew nut shell liquid revealed through the GC/MS analysis the presence of cardanols, cardols, phytosterol, triacontanes and anacardic acid; it was applied for the DPPH test and, technical cashew nut shell liquid of 1000 g/mL reduced the radical level by 88.9%. Regarding the enzymatic assay of xanthine oxidase, for scavenging radical hydroxyls, it presented high antioxidant activity (IC_50_ = 702 µg/mL) [113].

Another study reported the presence of anacardic acids in cashew nut shell liquid and salicylic acids derivatives (monoene–15:1, diene–15:2 and triene–15:3) with a C15 chain and different unsaturations, which are related to the antioxidant capacity. Therefore, the most unsaturated chain had higher antioxidant activity in the DPPH assay and, on the other hand the less unsaturated chain was the most active against the fungi *Trychophyton rubrum* [65].

The volatile oil of species *A. occidentale* and *A. humile*, was evaluated for antibacterial activity against *S. aureus*, *B. cereus* and *E. coli,* where *A. occidentale* showed higher inhibition zones for Gram-positive bacteria than Gram-negative bacteria. It is important to reveal the majority content of the oil in *A. occidentale*, which has (*E*)-caryophyllene (15.4%) and germacrene D (11.5%) and, the content in *A. humile*, (*E*)-caryophyllene (31.0%) and *α*-pinene (22.0%). The mechanism of action suggested for this oil is the pro-oxidant damage that occurs within the bacterial cell membrane, inducing the lipid peroxidation and, it could explain the preservative predicate [71].

The terpenes found in essential oils are responsible for their biological properties, highlighting the antimicrobial activity and, they have been used against foodborne pathogens. Studies suggest that the oil is able to penetrate the cell membrane and to promote antibacterial activity, confirming the previous hypothesis [114].

The ethanol extract from stem bark of *A. occidentale* presented distinct compounds, such as fatty acids, sitosteryl esters derivatives, sitosterol, stigmasterol, stigmasterol-3-*O*-β-galactopyranoside, sitosterol-3-*O*-β-galactopyranoside, sitosterol- 3-*O*-β-glucopyranoside and a mixture of anacardic acids (monoene and diene); this extract showed potent antioxidant activity in DPPH assay [115].

Leaf extracts of *A. humile* rich in tannins presented antibacterial activity against *S. aureus, P. aeruginosa* and *E. faecalis* [116]. The species *A. microcarpum* exhibited antioxidant activity in the DPPH (IC_50_) assay from the ethyl acetate fraction (27.9) and ethanol fraction (32.9) of the stem bark and, also for lipid peroxidation induced by Fe^2+^ in rat brains and liver homogenates [117].

A review of antioxidant and antimicrobial activities of *A. occidentale* and *A. microcarpum* selecting aerial parts (leaves, fruit peels, fruit), stem bark and oil brings a plenty of information about diverse in vivo and in vitro tests evaluated in different antioxidant assays, DPPH, thiobarbituric acid reactive substances (TBARS), ABTS, TAC, superoxide dismutase (SOD), catalase (CAT), and, the DPPH test was the most used. The antimicrobial assay was performed against a diversity of microorganisms, bacteria (gram positive and negative) as like as: *S. aureus*; *B. subtilis*; *Salmonella enterica*; *Shigella* sp.; *E. coli*; *P. aeruginosa*; *P. mirabilis*; *B. subtilis*; *K. pneumoniae*; *Clostridium sporogens*; *Streptococcus* spp.; and fungi: *C. albicans* and *Candida pseudotropicalis* [5].

Observing the many reports of wide spectra of microorganism’s inhibition by *Anacardium* species, mainly *A. occidentale* presents plenty of great results for antioxidant activity due to its metabolites. Overall, this study strengthens the idea that the *Anacardium* genus is a potential agent for food preservation, being more sustainable and safer than synthetic preservatives, since it is consumed by humans [71,108,112,114].

## 6. Conclusions and Future Perspectives

The nutritional value of cashew nuts and the phytochemical composition of *Anacardium* spp. make these plants interesting for food preservation and potential therapeutic applications. 

Further efforts should be made to investigate applications of standardized *Anacardium* plants using well-designed studies owing to their widespread use. The advance in agronomic research can modulate the obtaining of *Anacardium* plants with a better and known phytochemical composition. Moreover, the use of *Anacardium* spp. in the food industry as a food preservative is an interesting approach to the development of green foods without synthetic antioxidant or preservatives. In fact, antioxidant and antimicrobial compounds present in *Anacardium* comprise a broad set of molecules that worth be exploited to improve overall quality and increase food shelf-life.

## Figures and Tables

**Table 1:** Amino acids present in *Anacardium occidentale*.

Country/Area	Amino Acid	Plant Part/Culture/Extract	References
Nigeria, India, Spain	arginine alanine aspartic acid cysteine/cystine glutamic acid glycine histidine isoleucine leucine lysine methionine phenylalanine proline serine threonine tryptophan tyrosin evaline	Good grade and discarded cashew nut meal, cashew nuts, whole and defatted cashew nut flours, Vietnamese, Indian (Kerala origin) Brazilian, and Ivory Coast cashew kernels	[17,20,21,22]

**Table 2:** Vitamins and functional biofactors present in *Anacardium occidentale*.

Variety/Cultivar	Country/Area	Vitamins/Functional Biofactors	Plant Part/Culture/Extract	References
-	Brazil Spain	vitamin C + dehydroascorbic acid)	fresh and processed apple juice, Vietnamese, Indian (Kerala origin) Brazilian, & Ivory Coast cashew kernels	[17,25]
M 6/1, Bla 256-1, M 10/4 and M 44/3, Red & Yellow fruited species	Nigeria	vitamin C	cashew apples and kernels, fruit, leaves, stem bark and roots	[24,26]
-	Spain	vitamins B1, B5 (pantothenic acid, microbiological) vitamin B6, B8 (biotin, microbiological), B9 (total folate, microbiological), and B12	Vietnamese, Indian (Kerala origin) Brazilian, and Ivory Coast cashew kernels	[17]
Red & Yellow fruited species	Nigeria Spain	vitamins B2 and B3	fruit, leaves, stem bark and roots, Vietnamese, Indian (Kerala origin) Brazilian, and Ivory Coast cashew kernels	[17,24]
-	Indonesia, Spain	vitamin E (tocopherol/α-tocopherol/γ-tocopherol/δ-tocopherol)	kernels, kernels of cashew nut, Vietnamese, Indian (Kerala origin) Brazilian, and Ivory Coast cashew kernels	[4,17,27]
-	Spain	vitamin K1	Vietnamese, Indian (Kerala origin) Brazilian, and Ivory Coast cashew kernels	[17]
-	India	m-digallic acid	flowers	[28]
-	India	ethylgullute methyl gallute leucocyanidin leucodelphinidin	leaves	[28]
-	Indonesia	lutein zeaxanthin	kernels	[4,27]
Red & Yellow fruited species	Indonesia, Nigeria	thiamine	kernels, fruit, leaves, stem bark and roots	[4,24,27]

**Table 3:** Mineral composition in *Anacardium occidentale.*

Country/Area	Mineral	Plant Part/Culture/Extract	Reference
Spain	calcium iron magnesium potassium phosphorus sodium zinc	Vietnamese, Indian (Kerala origin) Brazilian, and Ivory Coast cashew kernels	[17]

**Table 4:** Fatty acids and esters present in *Anacardium occidentale.*

Country/Area	Fatty Acids and Esters	Plant Part/Culture/Extract	References
Spain, Indonesia	C18:0 stearic acid	whole and defatted cashew nut flours, kernels, Vietnamese, Indian (Kerala origin) Brazilian, and Ivory Coast cashew kernels	[4,17,20,27]
Spain	saturated fatty acid C17:0 heptadecanoic acid C20:0 arachidic acid C22:0 behenic acid C24:0 lignoceric acid monounsaturated fatty acid C20:1 gadoleic acid polyunsaturated fatty acid C18:3n3 linolenic acid trans fatty acid C18:1n9t elaidic acid C18:1n7t vaccenic acid	Vietnamese, Indian (Kerala origin) Brazilian, and Ivory Coast cashew kernels	[17]
China	C18:1t *trans-*oleic acid	cashew nut shell liquid	[30]
India	β-sitosterol	leaves and shoot powder, tender leaves	[28,31]
India	stigmasterol	leaves and shoot powder	[31]
Nigeria	1-cyclohexylnonene 2-trifluoroacetoxydodecane 3-[(trimethylsilyl)oxy]-17-[o-(phenyl methyl)oxime]-(3α,5α)-androstan -11,17-dione 5-methylbut-2-en-1-yl 3-hydroxy-5-methoxy cyclohexane carboxylate *cis-*oleic acid cyclohexane carboxylic acid cyclohexanecarboxylic acid, decyl ester decyl ester	cracked bark	[32]

**Table 5:** Polysaccharides present in *Anacardium occidentale.*

Country/Area	Polysaccharides	Plant Part/Culture/Extract	Reference
Brazil	arabinose galactose glucose glucuronic acid mannose rhamnose	crude gum	[33]

**Table 6:** Phenolic and carotenoids composition in *Anacardium occidentale*.

Variety/Cultivar	Country/Area	Chemical Constituents	Plant Part/Extract	References
**Phenolic compounds**				
Elongated yellow, elongated red and rounded red	Brazil, Cameroon	2-hydroxy-6-pentadecylbenzoic acid	leaves, cashew apple, nuts	[37,38]
Elongated yellow, elongated red and rounded red	Brazil	2,6-dihydroxybenzoic acid	cashew apple	[37]
-	India	cyaniding peonidin	cashew nut shell liquid	[39]
-	India, Cameroon, Poland, Tanzania	anacardic acid	cashew nut shell liquid, cashew seed	[35,38,39,40]
-	India, Cameroon, Poland, Tanzania	cardanol (decarboxylated anacardic acid)	cashew nut shell liquid, nuts	[35,38,39,40,41]
-	India, Poland, Tanzania	cardol	cashew nut shell liquid	[35,39,40,41]
-	Cameroon	salicyclic acid	nuts	[38]
-	India	ethyl gallate	flowers	[28]
-	India	hyperoside (quercetin 3-galactoside)	leaves, flower	[28]
Yellow and red	Brazil, Cote d’Ivoire	gallic acid	cashew apple juice, juice, stem bark	[42,43,44]
-	Brazil	5-hydroxymethylfurfural cinnamic acid protocatechuic acid	cashew apple juice	[44]
-	Indonesia, Brazil	(+)-catechin	kernels of cashew nut, bark	[27,42]
Yellow and red	Cote d’Ivoire	caffeic acid coumaric acid ferulic acid naringenin	juice	[43]
-	Brazil	amentoflavone derivate	leaves	[45]
-	Jamaica	stigmast-4-en-3-one stigmast-4-en-3-ol	bark	[46]
Yellow and red	Brazil, Malaysia	myricetin-*O*-glycoside	cashew apple, young tender leaves	[47,48]
Yellow and red	Malaysia	kaempferol-3-*O*-arabinofuranoside kaempferol-3-*O*-arabinopyranoside kaempferol-3-*O*-xyloside quercetin-3-*O*-xyloside unknown quercetin pentose unknown quercetin conjugate	young tender leaves	[48]
Yellow and red	Brazil, Malaysia	kaempferol-3-*O*-glucoside quercetin 3-*O*-arabinofuranoside	cashew apple, freeze dried, young tender leaves	[47,48]
-	Colombia *	1,2,3-benzenetriol 2-methyl-2-propenoic acid 2,2′-methylenebis(6-(1,1-dimethylethyl)-4-ethyl-pheno 2,2′,6,6′-tetramethyl-4,4′-methylenediphenol hexadecane 2,4-dioctylphenol 2,6-di[p-cyanophenyl]-4-picoline 4-methyl)furan-3-carboxylate 9-octadecenoic acid,12-hydroxy-13- methyl ester methyl 2-[2-(2-ethyl-1,3-dioxolan-2-ylmethyl)-1-hydroxipent-4-enyl]-N-(benzyl) benzenesulfonamide	integument	[49]
-	Colombia *	2-(1,1-dimethylethyl)-4-(1,1,3,3-tetramethylbutyl)phenol	integument, extracts and fractions	[49]
-	Malasiya, Brazil	myricetin	cashew shoots, cashew apple	[47,50]
Yellow and red	India, Malasiya, Brazil, Cote d’Ivoire	quercetin	flowers, cashew shoots, leaves, cashew apple, juice	[28,43,45,47,50]
-	Brazil	3-*O*-hexoside of methyl-cyanidin 5-methylcyanidin chloride delphinidin chloride kaempferol rhamnetin *O*-glycoside quercetin *O*-glycoside	cashew apple	[47]
-	India Brazil	delphinidin	cashew nut shell liquid, cashew apple	[39,47]
-	Nigeria	agathisflavone	leaves	[51,52]
-	Nigeria	quercetin 3-*O*-rutinoside	leaves	[51]
Yellow and red	Brazil, Malaysia, Nigeria	quercetin 3-*O*-rhamnoside	cashew apple, freeze dried cashew apple, young tender leaves	[47,48,51]
-	Brazil	5-methylcyanidin 3-*O*-hexoside myricetin 3-*O*-arabinofuranoside myricetin 3-*O*-arabinopyranoside myricetin 3-*O*-galactoside myricetin 3-*O*-glucoside myricetin 3-*O*-xylopyranoside quercetin 3-*O*-xylo-pyranoside rhamnetin	cashew apple, freeze dried cashew apple	[47]
Yellow and red	Brazil, Malaysia	myricetin 3-*O*-rhamnoside quercetin 3-*O*-arabino-pyranoside quercetin 3-*O*-galactoside quercetin 3-*O*-glucoside	cashew apple, freeze dried, young tender leaves	[47,48]
-	Brazil, Costa Rica	quercetin 3-*O*-arabinopyrannoside	freeze dried cashew apple	[47]
Yellow, orange, and red	-	7-*O*-methylcyanidin 3-*O*-β-d-galactopyranoside	peeled cashew apples	[53]
-	Spain, Costa Rica	provitamin A	Vietnamese, Indian (Kerala origin) Brazilian, and Ivory Coast cashew kernels, yellow-, orange-, and red-peeled cashew apples	[17,53]
clones CCP 76 and EMBRAPA 50	Brazil	(-)-epicatechin (-)-epigallocatechin	cashew apples, stem bark	[42,54]
clones CCP	Brazil	(-)-epicatechin-3-*O*-gallate (-)-epigallocatechin-*O*-gallate	cashew apples	[54]
**Phenolic lipids**				
-	Poland, Tanzania	2-methyl cardol	cashew nut shell liquid	[35,40]
-	Brazil	lycopene	cashew apple	[55]
**Carotenoids, carotenoid esters**				
-	Indonesia, Brazil, Spain, Costa Rica	β-carotene	kernels, cashew apple, Vietnamese, Indian (Kerala origin) Brazilian, and Ivory Coast cashew kernels, yellow-, orange-, and red-peeled cashew apples	[4,17,27,53]
Yellow, orange, and red	Costa Rica	β-cryptoxanthin palmitate	peeled cashew apples	[53]

* *Anacardium excelsum*.

**Table 7:** Oil and volatile composition of *Anacardium occidentale.*

Variety/Cultivar	Country/Area	Chemical Constituents	Plant Part/Culture/Extract	References
Red cashew	Nigeria, Bénin Kenya	(*E*)-β-ocimene	leaves, oil, cashew leaves	[68,69,70]
Red cashew	Nigeria, Bénin Brazil	δ-cadinene	leaves, oil, aerial parts	[68,69,71]
Red cashew, yellow cashew	Nigeria Spain	palmitic acid	fruits, whole and defatted cashew nut flours, Vietnamese, Indian (Kerala origin) Brazilian, and Ivory Coast cashew kernels	[17,20,68]
Red cashew	Nigeria	palmitic oleic acid	fruits	[68]
Yellow cashew	Nigeria	4-hydroxydodecanoic acid lactone (*E*)-hex-2-enol (*Z*)-hex-3-enol	fruits	[68]
Red cashew	Nigeria	benzyl tiglate methyl salicylate	flowers	[68]
-	China	*trans-*linoleic acid	cashew nut shell liquid	[30]
-	Bénin Spain, Indonesia, Brazil	C18:2 linoleic acid	leaves oil, whole and defatted cashew nut flours, kernels of cashew nut, Vietnamese, Indian (Kerala origin) Brazilian, and Ivory Coast cashew kernels	[4,17,20,27,69]
-	China	*trans-*linolenic acid isomers 9 t,11 t-CLA and 10 t,12 t- CLA isomers	cashew nut shell liquid	[30]
-	Brazil	1-butanol 1-hexanol 1-pentanol 2-butoxy-ethanol 2-methyl-2-pentenal 2-methyl butanoic acid 2-hexanol 3-hexanol 3-hydroxy-2-butanone 3-methyl-1-butanol 3-methyl butanoic acid 4-ethyl benzaldehyde 6-methyl 5-hepten-2-one ethyl 2-hydroxy-4-methylpentanoate ethyl 3-methylbutanoate ethyl 3-methylpentanoate ethyl acetate ethyl butanoate ethyl propanoate ethyl *trans*-2-butenoate ethyl *trans*-2-hexenoate ethyl *trans*-3-hexenoate isoamyl acetate methyl 2-butenoate methyl 2-methylene butanoate methyl 2-hexenoate methyl 3-methyl butanoate methyl 3-methyl pentanoate methyl butanoate *N*-amyl acetate octanal *trans*-2-hexenal γ-hexalactone δ-octalactone	apple juice	[72]
Nanum	Brazil	benzalde hydeethyl pentanoate hexanal	cashew apple, apple juice	[72,73]
-	Brazil Bénin	cis-3-hexenol	apple juice, leaves oil	[69,72]
Nanum	Brazil Bénin	nonanal	cashew apple, apple juice, leaves oil	[69,72,73]
-	Brazil	acetic acid	apple juice, cashew nut, fruit, cashew nut, juice	[72,74]
Nanum red cashew	Brazil Nigeria, Bénin	*α* -copaene	cashew apple, leaves, leaves oil, aerial parts	[68,69,71,73]
-	Brazil	(*E*)-caryophyllene	aerial parts, leaves oil	[71]
-	Bénin, Brazil	ɑ-humulene *α*-muurolene β -selinene δ-selinene aromadendrene bicyclogermacrene germacrene B germacrene D	aerial parts, leaves oil	[69,71]
-	Brazil	*α*-amorphene β-camigrene δ-cadinol	aerial parts	[71]
Nanum	Bénin Brazil	ɑ -cadinol γ-cadinene	cashew apple leaves oil, aerial parts	[69,71,73]
-	Kenya	(*Z*)-ocimene alloocimene	cashew leaves	[70]
-	India	cardanol (C13) cardanol (C15) cardanol (C17) cardol diethyl phthalate dimethyl anacardate	nut shell liquid	[75]
-	Brazil	anacardic acid diene anacardic acid monoene anacardic acid triene cardanol diene cardanol triene cardol diene cardol monoene cardol triene	nut shell liquid	[65]
-	USA	6-n-C15 alkylsalicylic acid	nut shell	[76]
-	Nigeria.	agathisflavone	leaves	[77]
**Esters**				
Nanum	Brazil	butyl 2-methyl-2-butenoate ethyl 2-butenoate ethyl 2-hexenoate ethyl 2-hydroxy-4-methylpentanoate ethyl 2-methyl-2-butenoate ethyl 2-octenoate ethyl 3-methylbutanoate ethyl benzenoacetate ethyl decanoate ethyl hexadecanoate ethyl tetradecanoate hexyl 2-methyl-2-butenoate hexyl benzoatemethyl 2-methyl-2-butenoate methyl 3-methylbutanoate methyl benzoate pentyl isopentanoate *E*-ethyl 3-hexenoate *E*-ethyl cinnamate *E*-methyl cinnamate *Z*-ethyl 3-hexenoate *Z*-ethyl cinnamate	cashew apple	[73]
Nanum	Brazil	ethyl 2-methylbutanoate ethyl benzoate ethyl hexanoate ethyl octanoate methyl hexanoate	cashew apple, apple juice	[72,73]
**Lactones**				
Nanum	Brazil	γ -dodecalactone γ-nonalactone	cashew apple	[73]
**Carboxylic acids**				
Nanum	Brazil	C8:0 octanoic acid C9:0 nonanoic acid C10:0 decanoic acid C14:0 tetradecanoic acid C18:0 octadecanoic acid	cashew apple	[73]
Nanum	Brazil Spain	C12: 0 dodecanoic acid	cashew apple Vietnamese, Indian (Kerala origin) Brazilian, and Ivory Coast cashew kernels	[17,73]
Nanum	Brazil Bénin Nigeria Spain	C16: 0 hexadecanoic acid	cashew apple, leaves oil, aerial parts, cracked bark, Vietnamese, Indian (Kerala origin) Brazilian, and Ivory Coast cashew kernels	[17,32,69,71,73]
-	Nigeria	octadecanoic acid, 2,3-dihydroxypropyl ester	cracked bark	[32]
**Aldehydes and ketones**				
Yellow cashew Nanum	Nigeria, Brazil	furfural	fruits, cashew apple	[68,73]
Nanum	Brazil	6-methyl-5-hepten-2-one acetophenone decanal phenylacetaldehyded *E*-2-decenal	cashew apple	[73]
**Alcohols**				
Nanum	Brazil	l-octanol	apple juice, cashew apple	[72,73]
Yellow cashew, Nanum	Nigeria, Brazil	hexadecanol	flowers, cashew apple	[68,73]
Nanum	Brazil	octadecanol	cashew apple	[73]
**Terpenes**				
Nanum	Brazil	6-cadinene curcufenol geranylketone norisoprenoids o-cymene ɑ-calacorene ɑ-fram-bergamotene ɑ-muurulol ɑ-tujene β-caryophyllene γ-muurulene	cashew apple	[73]
	Bénin	p-cymenene *trans*-ɑ-bergamotene	leaves oil	[69]
Nanum	Brazil, Bénin	ɑ-cubebene β-bisabolene *cis*-ɑ-bergamotene	cashew apple, leaves oil	[69,73]
**Hydrocarbons**				
Nanum	Brazil	eicosane heptadecane hexadecane naphthalene nonadecane octadecane pentadecane tridecane	cashew apple	[73]
Nanum	Brazil	tetradecane	cashew apple, aerial parts	[71,73]
EMBRAPA	Brazil	1-O-*trans-*cinnamoyl-D-glucopyranose	cashew apple juice	[54]
-	Brazil Nigeria Bénin	ɑ-pinene	cashew nut juice, fresh leaves oil, leaves oil	[69,74,78]
-	Brazil Bénin	β-pinene	cashew nut, juice, leaves oil	[69,74]
Nanum	Bénin, Nigeria Brazil	limonene	cashew apple, fresh leaves oil leaves oil, aerial parts	[69,71,73,78]
-	Bénin	1-epi-cubenol 1-octen-3-ol 1,2-benzene dicarboxylic acid 1,4-dimethylbenzene 1,8-cineole 1,10-diepi-cubenol 2-nonadecanone 3-carene 4a-H,10a-H-guaia-1(5)-6-diene 4b-H,10a-H-guaia-1(5)-6-diene 5-epi-neointermedeol 6,10,14-trimethyl-2-pentadecanone 7,9-diterbutyl-1-oxaspiro-[4,5]-deca-6,9-diene-2,8-dione 7-octadiene-3,6-diol 9-epi-β-caryophyllene 10-epi-cubenol 10-epi-γ-eudesmol 16-kaurene allo-aromadendrene aristolone benzyl benzoate benzyl salycilate borneol bornyl acetate cadina-1,4-diene cadina-3,5-diene camphene camphor carvenone caryophyllene alcohol caryophyllene oxide citronellal citronellol *cis*-calamenene *cis*-linalool oxide *cis*-*p*-menth-2-en-1-ol chavicol cryptone cubenol cuminaldehyde cyclohexadecane dihydroedulan I dodecanophenone elemol epi-globulol epi-ɑ-cadinol epi-ɑ-muurolol eremophilene ethylbenzene farnesol geraniol geranyl formate germacrene A globulol guaia-6,9-diene hexyl tiglate humulene epoxide II intermedeol juniper camphor linalool *m*-mentha-1(7),8-diene manoyl oxide mentha-1,4-dien-7-ol menthone methyl chavicol methyl eugenol methyl linolenate mesitylene mintsulfide myrcene *p*-anisaldehyde *p*-cymen-7-ol *p*-cymene *p*-menth-1-en-9-ol *p*-mentha-1,5-dien-7-ol *p*-mentha-1,5-dien-8-ol *p*-mentha-1(7),2-dien-8-ol *p*-mentha-1(7),4(8)-diene piperitone phellandral phytol sabinene santolina alcohol salvial-4(14)-en-1-one selina-3,5-diene selina-3,7(11)-diene selina-4(15),7(11)-diene spathulenol terpinen-4-ol terpinolene thymol *trans*-calamenene *trans*-carveol *trans*-2-cyclohexen-1-ol *trans*-linalool oxide *trans*-*p*-menth-2-en-1-ol *trans*-piperitol *trans*-phytol *trans*-sabinol valencene viridiflorol ylanga-2,4(15)-diene zonarene ɑ-cadinene ɑ-fenchol ɑ-fenchyl acetate ɑ-muurolol ɑ-phellandrene ɑ-terpinene ɑ-terpineol ɑ-thujene ɑ-selinene β-acoradiene β-bourbonene β-bulnesene β-cedrene β-cubebene β-elemene β-phellandrene β-terpineol δ-amorphene δ-elemene γ-2-cadinene γ-amorphene γ-elemene γ-gurjunene γ-muurolene γ-pinocarveol γ-terpinene (*E*)-2-decenal 2,6-dimethyl-1 (*E*)-3-hexenyl butyrate (*E*)-3-hexenyl isovalerate (*E*)-β-farnesene (*E*)-β-ionone (*E*,*E*)-ɑ-farnesene (*Z*)-3-hexenyl-2-methyl butyrate (*Z*)-3-hexenyl benzoate (*Z*)-3-hexenyl isovalerate (*Z*)-3-hexenyl (*Z*)-3-hexenoate	leaves oil	[69]
-	Bénin, Kenya	(*Z*)-3-hexenyl butyrate	leaves oil, cashew leaves	[69,70]
-	Nigeria Bénin	ɑ-ylangene	fresh leaves oil, leaves oil	[69,78]
-	Brazil	2-furan-methanol butanoic acid	cashew nut, fruit, cashew nut juice	[74]
-	Brazil	1,3-dihydroxy-2-propanone 2-octil-cyclopropaneoctanal acetic acid 1-butanol ester acetic acid 2-propen-1-ol ester acetic acid 3-hexen-1-ol ester amfibina benzoic acid butanoic acid ethyl ester canfene hexanoic acid 2-propenyl ester hexanoic acid ethyl ester oxalic acid ammonium ester pentanoic acid 3-methylbutyl ester pentanoic acid ethyl ester sulphuric dioxide *trans-*caryophyllene	cashew nut, juice	[74]
-	Nigeria	17-octadecynoic acid rostane steroid 3-[(trimethylsilyl)oxy]-17-[o-(phenylmethyl)oxime]-(3α,5α)-androstane-11,17-dione	cracked bark	[32]

**Table 8:** Oil constituents in other species of the Anacardeaceae family.

Species	Variety/Cultivar	Country/Area	Chemical Constituents	Plant Part/Culture/Extract	Reference
**Hydrocarbon**					
*A. fraxinifolium*	-	Brazil	myrcene *α*-terpinolene *δ*-2-carene (*E*)-*β*-ocimene (*Z*)-*β*-ocimene	aerial parts	[71]
*A. fraxinifolium* *A. humile*	-	Brazil	*α*-pinene	aerial parts	[71]
*A. humile*	-	Brazil	*β*-pinene	aerial parts	[71]
*A. fraxinifolium A. humile* *A. occidentale*	-	Brazil	limonene	aerial parts	[71]
**Hydrocarbon Sesquiterpenes**					
*A. fraxinifolium*	-	Brazil	viridiflorene *α*-amorphene (*E*,*E*)-α-farnesene	aerial parts	[71]
*A. fraxinifolium* *A. humile*	-	Brazil	alloaromadendrene aromadendrene germacrene D *α*-copaene *γ*-cadinene	aerial parts	[71]
*A. humile*	-	Brazil	bicyclogermacrene germacrene A *α*-gurjunene *α*-humulene *α*-muurolene *β*-selinene *δ*-cadinene (*E*)-caryophyllene	aerial parts	[71]
*A. occidentale*	-	Brazil	*β*-camigrene	aerial parts	[71]
**Oxygenated Sesquiterpenes**					
*A. fraxinifolium*	-	Brazil	ledol spathulenol	aerial parts	[71]
*A. fraxinifolium* *A. humile*	-	Brazil	globulol viridiflorol	aerial parts	[71]
*A. humile*	-	Brazil	epiglobulol *α*-cadinol *β*-caryophyllene oxide	aerial parts	[71]
*A. humile*	-	Brazil	*α*-pinene (*E*)-caryophyllene	leaves oil	[71]

**Table 9:** Other phytochemicals present in *Anacordium occidentale*.

Country/Area	Chemical Constituents	Plant Part/Extract	References
Indonesia	2-methyl-5-pentadecylresorcinol 2-methyl-5[8(2), 11(2)-pentadecadienyll resorcinol 2-methyl-5[8(2), 11(2),14-pentadecatrienyll resorcinol 2-methyl-5[8(*Z*)-pentadecenyl] resorcinol3-pentadecylphenol 3-[8(2), 11 (2)-pentadecadienyll-phenol 3-[8(*Z*)-pentadecenylIphenol 3-18(2), 1 1(2), 14-pentadecatrienyllphenol 5-pentadecylresorcinol5-[8(2), 11(2)-pentadecadienyllresorcinol 5-[8(2), 11(*Z*),1 4-pentadecatrienyllresorcinol 6-pentadecylsalicylic acid 6-[8(2), 11 (21,14-pentadecatrienyllsalicylic acid 6-[8(*Z*)-pentadeccnyl]salicylic acid 6-18(2), 11(2)-pentadecadienyl]salicylic acid 14,5-[8(*Z*)-pentadecenyl]-resorcinol	cashew nut shell	[86]
Brazil	anacardic acid	fruit extracts, fruit	[87,88,89,90]
Brazil	acid 6-pentadec(en) salicylic	fruit	[90]
Brazil	alkyl phenols cardols	fruit	[87]
Brazil	acid 6-alqu(en) ilsalicylic	bark extract, fruit	[91]
Brazil	cardanols	fruit extracts, fruit	[87,88,89]
Brazil	cardol 2-methyl-cardol	fruit extracts	[88]
Colombia *	bi flavonoids	total extract	[92]
Colombia **	acid 6-alkilsalic alkyl resorcinols	fruit extracts	[93]
India	occidentoside β-sitosterol	nut shells	[60]
Hong Kong	2-alkylcyclobutanones	cashew nut samples	[94]
**Cytotoxic compounds**			
Nigeria	anacardicin zoapatanolide A [1,2-bis(2,6-dimethoxy-4-methoxycarbonylphenyl)ethane] methyl gallate	leaves	[52]

* *A. humile*; ** *A. giganteum*.
